# Population Pharmacokinetic Properties of Piperaquine in Falciparum Malaria: An Individual Participant Data Meta-Analysis

**DOI:** 10.1371/journal.pmed.1002212

**Published:** 2017-01-10

**Authors:** Richard M. Hoglund, Lesley Workman, Michael D. Edstein, Nguyen Xuan Thanh, Nguyen Ngoc Quang, Issaka Zongo, Jean Bosco Ouedraogo, Steffen Borrmann, Leah Mwai, Christian Nsanzabana, Ric N. Price, Prabin Dahal, Nancy C. Sambol, Sunil Parikh, Francois Nosten, Elizabeth A. Ashley, Aung Pyae Phyo, Khin Maung Lwin, Rose McGready, Nicholas P. J. Day, Philippe J. Guerin, Nicholas J. White, Karen I. Barnes, Joel Tarning

**Affiliations:** 1 WorldWide Antimalarial Resistance Network, Oxford, United Kingdom; 2 Mahidol Oxford Tropical Medicine Research Unit, Faculty of Tropical Medicine, Mahidol University, Bangkok, Thailand; 3 Centre for Tropical Medicine and Global Health, Nuffield Department of Medicine, University of Oxford, Oxford, United Kingdom; 4 Division of Clinical Pharmacology, Department of Medicine, University of Cape Town, Cape Town, South Africa; 5 Department of Drug Evaluation, Australian Army Malaria Institute, Brisbane, Queensland, Australia; 6 Department of Malaria, Military Institute of Hygiene and Epidemiology, Hanoi, Viet Nam; 7 Department of Infectious Diseases, Military Hospital 108, Hanoi, Viet Nam; 8 Institut de Recherche en Sciences de la Santé, Direction Régionale de l’Ouest, Bobo-Dioulasso, Burkina Faso; 9 London School of Hygiene & Tropical Medicine, London, United Kingdom; 10 Kenya Medical Research Institute–Wellcome Trust Research Programme, Kilifi, Kenya; 11 Institute for Tropical Medicine, University of Tübingen, Tübingen, Germany; 12 Joanna Briggs Affiliate Centre for Evidence-Based Health Care, Evidence Synthesis and Translation Unit, Afya Research Africa, Nairobi, Kenya; 13 Global and Tropical Health Division, Menzies School of Health Research and Charles Darwin University, Darwin, Northern Territory, Australia; 14 Department of Bioengineering and Therapeutic Sciences, University of California San Francisco, San Francisco, California, United States of America; 15 Yale School of Public Health and Medicine, New Haven, Connecticut, United States of America; 16 Shoklo Malaria Research Unit, Faculty of Tropical Medicine, Mahidol University, Mae Sot, Thailand; ISGlobal, SPAIN

## Abstract

**Background:**

Artemisinin-based combination therapies (ACTs) are the mainstay of the current treatment of uncomplicated *Plasmodium falciparum* malaria, but ACT resistance is spreading across Southeast Asia. Dihydroartemisinin-piperaquine is one of the five ACTs currently recommended by the World Health Organization. Previous studies suggest that young children (<5 y) with malaria are under-dosed. This study utilised a population-based pharmacokinetic approach to optimise the antimalarial treatment regimen for piperaquine.

**Methods and Findings:**

Published pharmacokinetic studies on piperaquine were identified through a systematic literature review of articles published between 1 January 1960 and 15 February 2013. Individual plasma piperaquine concentration–time data from 11 clinical studies (8,776 samples from 728 individuals) in adults and children with uncomplicated malaria and healthy volunteers were collated and standardised by the WorldWide Antimalarial Resistance Network. Data were pooled and analysed using nonlinear mixed-effects modelling. Piperaquine pharmacokinetics were described successfully by a three-compartment disposition model with flexible absorption. Body weight influenced clearance and volume parameters significantly, resulting in lower piperaquine exposures in small children (<25 kg) compared to larger children and adults (≥25 kg) after administration of the manufacturers’ currently recommended dose regimens. Simulated median (interquartile range) day 7 plasma concentration was 29.4 (19.3–44.3) ng/ml in small children compared to 38.1 (25.8–56.3) ng/ml in larger children and adults, with the recommended dose regimen. The final model identified a mean (95% confidence interval) increase of 23.7% (15.8%–32.5%) in piperaquine bioavailability between each piperaquine dose occasion. The model also described an enzyme maturation function in very young children, resulting in 50% maturation at 0.575 (0.413–0.711) y of age. An evidence-based optimised dose regimen was constructed that would provide piperaquine exposures across all ages comparable to the exposure currently seen in a typical adult with standard treatment, without exceeding the concentration range observed with the manufacturers’ recommended regimen. Limited data were available in infants and pregnant women with malaria as well as in healthy individuals.

**Conclusions:**

The derived population pharmacokinetic model was used to develop a revised dose regimen of dihydroartemisinin-piperaquine that is expected to provide equivalent piperaquine exposures safely in all patients, including in small children with malaria. Use of this dose regimen is expected to prolong the useful therapeutic life of dihydroartemisinin-piperaquine by increasing cure rates and thereby slowing resistance development. This work was part of the evidence that informed the World Health Organization technical guidelines development group in the development of the recently published treatment guidelines (2015).

## Abstract 2

Joel Tarning and colleagues combine individual participant data from clinical trials to develop a pharmacokinetic model and derive an optimized dose regimen for malaria treatment in children.

## Author Summary

**Why Was This Study Done?:**

- Despite expansion of malaria prevention and treatment in the last decade, malaria still kills around 1,200 people each day, mostly children below the age of five.
- Reduced drug exposure to one of the commonly used antimalarial drug combinations, dihydroartemisinin-piperaquine, has been reported in small children.
- It is crucial to develop an optimised dose regimen that achieves similar drug exposure in all patient groups, in order to give all patients an equal chance of cure.

**What Did the Researchers Do and Find?:**

- Drug concentration–time data (8,776 piperaquine concentration measurements) from 728 individuals in 11 separate clinical trials were collated and pooled for an individual participant data meta-analysis.
- A pharmacokinetic model was developed to describe the pharmacological properties of piperaquine, the expected variability between patients, and the influence of biologically important covariates.
- Small children had a substantially lower piperaquine exposure after recommended dosing regimens.
- The developed pharmacokinetic model was used to derive a new optimised dose regimen.

**What Do These Findings Mean?:**

- The proposed improved dose regimen of dihydroartemisinin-piperaquine is expected to provide equivalent piperaquine exposures safely in all patients, including in small children with malaria.
- An optimised dose regimen should prolong the useful therapeutic life of dihydroartemisinin-piperaquine by increasing cure rates and thereby slowing resistance development.

## Background

Malaria currently causes an estimated 1,200 deaths each day [1]. Most malaria-related deaths occur in Africa in children under the age of 5 y. In endemic areas, young children lack sufficient acquired immunity and are more likely to develop severe forms of the disease. Artemisinin-based combination therapy (ACT) is the recommended first-line treatment for uncomplicated *Plasmodium falciparum* malaria. The 3-d fixed-dose combination of dihydroartemisinin and piperaquine is one of five ACTs currently recommended by the World Health Organization (WHO) [2]. The rapidly eliminated dihydroartemisinin component has a very potent antimalarial effect and eliminates the majority of the parasite biomass during the first 3 d of treatment [3]. The partner drug, piperaquine, is a slowly eliminated antimalarial that kills the residual parasites that remain after two asexual life cycles of exposure to dihydroartemisinin, thereby preventing recrudescent malaria. Piperaquine also prevents reinfections for approximately 1 mo after treatment [4–11]. The principal determinant of the therapeutic response of a slowly eliminated antimalarial drug is the duration for which the plasma (and thus free drug) level exceeds the minimum inhibitory concentration, which is reflected by the area under the plasma concentration–time curve, or its surrogate, the day 7 level [12].

Although there are several producers of dihydroartemisinin-piperaquine, three main manufacturers are producing and distributing dihydroartemisinin-piperaquine in endemic countries: Sigma-Tau Pharmaceuticals produces Eurartesim, registered with the European Medicine Agency in 2012; Guilin Pharmaceutical produces D-Artepp; and Beijing Holley-Cotec Pharmaceuticals produces Duo-Cotexin. Sigma-Tau’s recommendation is a target daily dosage of 18 mg piperaquine phosphate per kilogram body weight across all age groups, with a practical weight-based dosing schedule provided [13]. Beijing Holley-Cotec provides two weight-based dosing schedules, one for children, with a target daily dosage of 16 mg/kg, and one for adults [14,15]. Both manufacturers’ dosage recommendations are based on evidence from the early stages of piperaquine development before there was extensive information on the pharmacokinetic properties of piperaquine in young children (<5 y of age) and before resistance to artemisinins was established. Artemisinin resistance results in lower fractional reductions in parasite numbers per asexual cycle, leaving a larger residual biomass of parasites for the partner drug to remove. This increases the probability of recrudescence and drives the spread of resistance. First artemisinin, and now piperaquine, resistance has emerged in Cambodia [16–18]. Elsewhere the dihydroartemisinin-piperaquine combination has shown excellent efficacy and tolerability, although young children treated with dihydroartemisinin-piperaquine have a 3-fold greater risk of recrudescent malaria compared with older children and adults [19–21]. Piperaquine is highly bound to plasma proteins (>98%), with a very large volume of distribution (>100 l/kg), a low hepatic elimination clearance (<1.4 l/h/kg), and a consequently long terminal plasma elimination half-life (estimates range from 18 to 28 d) [22–28]. The pharmacokinetic properties of piperaquine are affected by body weight, pregnancy, and age [24,25,27,29,30]. A large quantity of co-administered fat enhances absorption significantly, particularly in healthy volunteers, whereas a small amount of fat does not [25,31,32]. Previous reports on the pharmacokinetic properties of piperaquine in children are conflicting [22–24,33]. The larger studies indicated that small children have an inadequate plasma exposure to piperaquine after standard dosing, which led to a proposed increased dose regimen of dihydroartemisinin-piperaquine in order to achieve adequate exposures in small children [24,30].

Pharmacokinetic studies are often small and so have limited power to detect important covariates. Provided the assay performances are comparable, pooling of individual participant data from several studies increases the power to determine covariates with higher precision and accuracy. Nonlinear mixed-effects modelling for pharmacokinetic meta-analyses permits a unifying structural, covariate, and statistical model to be developed [34]. Even with the best of tools, however, the heterogeneity of study designs and assay methods makes these analyses challenging.

The WorldWide Antimalarial Resistance Network (WWARN) is a unique data sharing platform providing scientists and clinical investigators with an opportunity to share their data, knowledge, and experience. The aim of this study was to use pooled individual participant pharmacokinetic data from WWARN to characterise the pharmacokinetic properties of piperaquine, with a special focus on small children. Stochastic simulations from the final model were used to develop an evidence-based optimised dose regimen based on maximum concentration (as a measurement of toxicity) and piperaquine concentration at day 7 (as a measurement of efficacy) [35].

## Methods

### Ethical Approval

Participating investigators agreed to the WWARN terms of submission [36], which ensure that all data uploaded are anonymized and obtained with informed consent, and in accordance with any laws and ethics committee approvals applicable in the country of origin. Ethics committee approval for the pooled analysis of individual participant data was granted by the Oxford Tropical Research Ethics Committee.

### Clinical Studies

**Fig 1:**
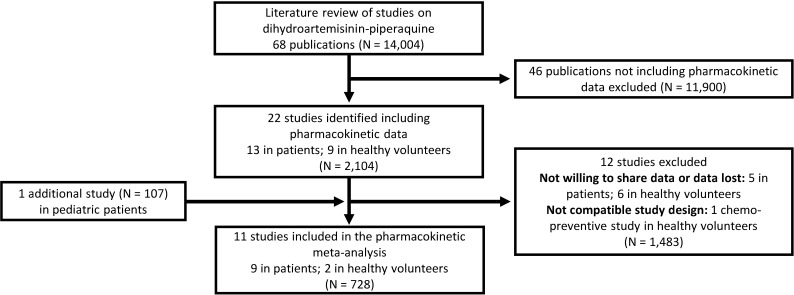
Flowchart of the literature search.

All published pharmacology studies reported in PubMed, Google Scholar, Embase, ClinicalTrials.gov, or conference proceedings were identified through a systematic literature review of articles published between 1 January 1960 and 15 February 2013 according to PRISMA guidelines (Fig 1); the PRISMA checklist can be found in S1 PRISMA IPD Checklist. Principal investigators were invited to contribute individual patient data to the WWARN repository as part of a study group conducting a collaborative pooled analysis provided that their studies met the following criteria: (i) prospective dihydroartemisinin-piperaquine study in patients with uncomplicated *P*. *falciparum* infection or in healthy volunteers and (ii) validated measure of capillary and/or venous plasma piperaquine concentrations available. All data were uploaded to the WWARN repository and standardised using a methodology described in the WWARN clinical and pharmacology data management and statistical analysis plans [37,38]. Study reports generated from the formatted datasets were sent back to investigators for clarification and/or validation. Pharmacokinetic data from ten previously published clinical studies and one unpublished (at the time) clinical study were contributed and used for modelling [22,24,25,27,29,30,39–43]. Demographic data from each study are summarised in Table 1. Study protocols for the studies were available in the original publication or on request from the data contributor. Individual-patient-level data are available through WWARN (http://www.wwarn.org). Requests for access will be reviewed by a Data Access Committee to ensure that use of data is within the terms of consent and ethics approval. WWARN is registered with the Registry of Research Data Repositories (http://re3data.org).

**Table 1:** Demographic data of study population for the pooled analysis.

Population	Country	Number of Individuals	Weight (kg)	Age (Years)	Female (Percent)	Pregnant (Percent)	Malaria Infection	Dose Regimen	Number of Samples	Median (Range) Number of Samples per Individual	Venous or Capillary Measurement	Ethical Approval	Reference
Pregnant and non-pregnant women	Thailand	48	36–78	18–45	100	50	Pf	3 doses over 3 d	1,250	33 (23–34)	Venous	MUTM (2007–111), OxTREC (017–07)	[27]
Pregnant and non-pregnant women	Sudan	24	44–81	16–43	100	50	Pf	3 doses over 3 d	589	26 (9–30)	Venous	National College for Medical and Technical Studies (Khartoum)	[44]
Adults	Viet Nam	18	31–55	16–49	33.3	0	Pf	2 doses over 2 d	18	1	Venous	Viet Nam People’s Army Department of Military Medicine, ADHREC (No. 507/08)	[43]
Adults	Thailand	30	39–73	18–55	13.3	0	Pf	3 doses over 3 d	1,038	36 (27–37)	Venous	OxTREC, ECFTM	[25]
Adults	Viet Nam	12	48–75	19–51	0	0	Pf	3 doses over 3 d	240	20	Venous	Review and Scientific Board of Military Hospital 175, ADHREC (No. 379/05)	[42]
Children and adults	Thailand	98	12–74	3–55	39.8	0	Pf	3 or 4 doses over 3 d	482	5 (3–10)	Venous	ECFTM, OxTREC	[22]
Children	Burkina Faso	236	8–34	2.0–10	44.7	0	Pf	3 doses over 3 d	2,471	11 (4–12)	Capillary	CEIM, CHRUC	[24]
Children	Kenya	105	6.3–21	0.58–4.9	56.2	0	Pf	3 doses over 3 d	105	1	Venous	NKEMRIERC, OxTREC, ECHUSM	[39]
Healthy adults	Viet Nam	24	56–70	18–29	0	0	Healthy	Single dose	824	17 (16–18)	Venous	Review and Scientific Board of Central Military Hospital 108, ADHREC (No. 437/06)	[41]
Healthy adults	Viet Nam	26	54–70	19–24	0	0	Healthy	Single dose and 3 doses over 3 d	495	19.5 (18–20)	Venous	Review and Scientific Board of Central Military Hospital 108, ADHREC (No. 361/04)	[40]
Children	Uganda	107	5.1–12	0.56–1.8	40.2	0	Pf	3 doses over 3 d	1,264	12 (4–25)	Capillary	UNCST, MUREC, UCSFCHR (#10–01881), CDCPGAIDSP (#5145.0)	[30]
NA	NA	728	5.1–81	0.56–55	43.3	4.9	NA	NA	8,776	17 (1–37)	NA	NA	NA

Female (percent) represents the proportion of females in the study. Pregnant (percent) represents the proportion of pregnant women of the total population in the study. Malaria infection indicates whether the individuals were healthy or had an uncomplicated *P*. *falciparum* (Pf) malaria infection.

ADHREC, Australian Defence Human Research Ethics Committee (Australia); CDCPGAIDSP, Centers for Disease Control and Prevention Global AIDS Program; CEIM, Comité d’Ethique Institutionnel du Centre Muraz (Burkina Faso); CHRUC, Committee on Human Research of the University of California (US); ECFTM, Faculty of Tropical Medicine Mahidol University Ethical Committee (Thailand); ECHUSM, Ethics Committee of the Heidelberg University School of Medicine (Germany); MUREC, Makerere University Research and Ethics Committee; MUTM, Faculty of Tropical Medicine Mahidol University Ethical Committee (Thailand); NA, not applicable; NKEMRIERC, National KEMRI Ethical Review Committee (Kenya); OxTREC, Oxford Tropical Research Ethics Committee (UK); UCSFCHR, University of California San Francisco Committee on Human Research; UNCST, Uganda National Council for Science and Technology.

### Pharmacokinetic Analysis

Plasma piperaquine concentrations (base form), transformed into their natural logarithms, were analysed using nonlinear mixed-effects modelling implemented in NONMEM v7.3 (ICON Development Solutions) with the first-order conditional estimation method [45,46]. Perl-Speaks-NONMEM 3.5.3, R v2.14.2 (R Foundation for Statistical Computing) with the Xpose package v4.3.5, and Piraña v2.6.0 were used for diagnostics and automation throughout the modelling process [47–49].

Piperaquine was administered as piperaquine phosphate, which was converted to piperaquine base with a scale factor of 57.7%. Two-, three-, and four-compartment disposition models were evaluated with first-order absorption. The best performing model was used to evaluate the most appropriate absorption model. First-order absorption with and without lag time and a more flexible transit absorption model with a fixed number (1–8) of transit compartments were investigated. Inter-individual variability was added exponentially to all parameters according to Eq 1:

$$P_{i}=\theta_{p}∙e^{\eta_{i,p}}$$ (1)

where *P*_*i*_ is the individual parameter estimate for the *i*th individual, θ_*p*_ is the population value of the investigated parameter, and η_*i*,*p*_ is the individual deviation from the population parameter value for the *i*th individual. The η is drawn from a normal distribution with mean zero and variance ω^2^ (diagonal correlation matrix). The bioavailability was fixed to unity for the population, though inter-individual variability of this parameter was allowed. Inter-occasion variability was evaluated on absorption parameters (i.e., bioavailability and mean transit time) to allow variability in rate and amount of piperaquine absorption between dosing occasions:

$$P_{i,j}=\theta_{p}∙e^{\eta_{i,p}+\kappa_{j,p}}$$ (2)

where *P*_*i*,*j*_ is the individual estimate of the investigated parameter at the *j*th dosing occasion for individual *i*. κ_*j*,*p*_ is the deviation from the population parameter value for the *j*th dose. The κ is drawn from a normal distribution with mean zero and variance Π^2^. The unknown variability in concentration was described by an additive error on the individually predicted logarithmic concentrations (i.e., equivalent to an exponential error on non-transformed concentrations).

Body weight was evaluated by adding it as an allometric function to all clearance (power of 0.75) and volume of distribution (power of 1) parameters before any other covariates were investigated, but an attempt was also made to estimate these exponents. The maturation process of enzyme-dependent biotransformation pathways in infants and its effect on elimination clearance in children below the age of 5 y was evaluated according to Eq 3:

$${CL}_{i}=\theta_{CL}∙\frac{{AGE}_{i}^{Hill}}{{MF}_{50}^{Hill}+{AGE}_{i}^{Hill}}∙e^{\eta_{i,CL}}$$ (3)

where CL_*i*_ is the individual clearance parameter estimate for the *i*th individual, θ_CL_ is the population value of the elimination clearance parameter, AGE_*i*_ is the individual’s age, MF_50_ is the age that results in 50% maturation, and Hill is the Hill coefficient describing the slope of the maturation process.

Disease effect and gender (i.e., sex) were evaluated as proportional categorical covariates in a subset of data to avoid false positive/negative relationships resulting from correlated covariates. Disease effect was evaluated on all parameters in a dataset with only adult (>18 y of age) male patients and healthy volunteers. The effect of gender was investigated on all parameters in malaria-infected non-pregnant adults. Dosing occasion as a categorical covariate for absorption parameters was investigated using all the available data. Total daily dose per body weight was also investigated as a linear covariate for absorption parameters. Substantial systematic differences in matched venous and capillary plasma piperaquine concentrations have been reported in a previous clinical study [50]. A proportional scaling factor between venous and capillary concentrations was therefore estimated to allow fitting of all data simultaneously:

$$C_{CAP}=C_{VEN}+\theta_{S}∙C_{VEN}$$ (4)

where *C*_CAP_ is the individually predicted capillary concentration, *C*_VEN_ is the individually predicted venous concentration, and θ_S_ is the population scale parameter between the two biological matrices.

Model discrimination was based on the objective function value (OFV) proportional to −2 times the log likelihood of data. A reduction in OFV of 3.84 and 10.8 was considered significant at *p =* 0.05 and *p =* 0.001, respectively, for a nested model with one degree of freedom difference. All covariates, except the allometric function of weight, were analysed in a step-wise manner with a forward selection step (*p =* 0.05) and a stricter backward elimination step (*p =* 0.001). Model diagnostics and predictive performance were evaluated by goodness-of-fit plots and simulation-based diagnostics (i.e., non-corrected, prediction-corrected, and variability-corrected visual predictive checks), respectively [51]. Parameter precision was investigated by generating 1,000 resampled datasets, stratified by clinical study, in a bootstrap approach. Parameter shrinkages were calculated [52] to determine the reliability of diagnostic plots.

### Dose Optimisation

Stochastic simulations of the final mixed-effects model were performed to evaluate the exposure to piperaquine after (i) the dose regimen recommended by Sigma-Tau, (ii) the dose regimen recommended by Beijing Holley-Cotec, and (iii) a putative optimised dose regimen, as presented in Table 2 [24]. A total of 1,000 malaria-infected non-pregnant patients were simulated per kilogram of body weight (range: 5 to 100 kg) and dose regimen. As a simple surrogate of total exposure, predicted day 7 venous plasma piperaquine concentrations were compared between simulations, while the maximum concentrations were compared as an indicator of possible acute toxicity.

**Table 2:** Evaluated dose regimens for piperaquine simulations.

Dose Regimen	Body Weight (kg)	Number of Tablets/Day	PQP/Day (mg/kg)	Total Dosage (mg/kg) by Body Weight Group					
				All		5–15 kg		>15 kg	
				Minimum	Maximum	Minimum	Maximum	Minimum	Maximum
**Sigma-Tau**				38.4	80.1	39.9	73.8	38.4	80.1
	5–6	0.25	13.3–16.0						
	7–12	0.5	13.3–22.9						
	13–23	1	13.9–24.6						
	24–35	2	18.3–26.7						
	36–74	3	13.0–26.7						
	75–100	4	12.8–17.1						
**Beijing Holley-Cotec**				28.8	96	39.9	68.7	28.8	96
	5–6	0.25	13.3–16.0						
	7–9	0.5	17.8–22.9						
	10–14	0.5*	15.2–21.3						
	15–19	1	16.8–21.3						
	20–39	2	16.4–32.0						
	40–100	3	9.60–24.6						
**Optimised**				48	96	64	96	48	84.7
	5–7	0.5	22.9–32.0						
	8–10	0.75	24.0–30.0						
	11–16	1	20.0–29.1						
	17–24	1.5	20.0–28.2						
	25–35	2	18.3–25.6						
	36–59	3	16.3–26.7						
	60–79	4	16.2–21.3						
	80–100	5	16.0–20.0						

PQP/day is the total daily dosage of piperaquine phosphate (mg/kg). Minimum total dosage is the minimum total piperaquine dosage an individual receives with each dose regimen. Maximum total dosage is the maximum total piperaquine dosage an individual receives with each dose regimen.

*One tablet given on the first day and half a tablet on the second and third day.

## Results

### Clinical Studies

A total of 11 different clinical studies were shared with WWARN, containing 8,776 plasma piperaquine concentrations from 728 individuals that could be included in the pooled analysis (Fig 1) [22,24,25,27,29,30,39–43]. Demographic data are presented in Table 1.

### Pharmacokinetic Properties of Piperaquine

Of the 8,776 samples included, 141 concentrations (1.74%) were measured to be below the lower limit of quantification. These were the only measurements omitted from the analysis.

**Fig 2:**
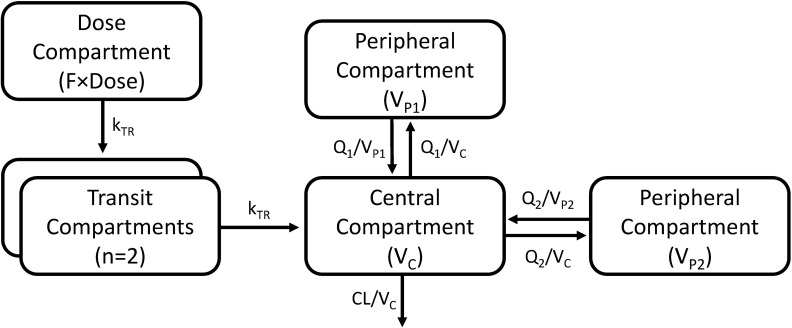
A graphical overview of the final piperaquine population pharmacokinetic model. *k*_TR_ is the absorption transit rate constant. CL is the elimination clearance. *V*_C_ is the volume of distribution of the central compartment. *V*_P1_ and *V*_P2_ are the volumes of distribution of the first and second peripheral compartments, respectively. *Q*_1_ and *Q*_2_ are the inter-compartment clearances for the first and second peripheral compartments, respectively. *F* is the relative oral bioavailability.

A three-compartment disposition model proved superior to a two-compartment disposition model (*p <* 0.001). There was no further improvement from an additional fourth compartment (*p* > 0.05). The absorption phase was described successfully by a transit compartment model with two transit compartments (*k*_A_ and *k*_TR_ were assumed equal). This model proved superior to all other tested absorption models (ΔOFV = −215). The addition of inter-individual variability in relative bioavailability improved the model fit significantly (*p <* 0.001). The final structural model is presented in Fig 2. Inter-individual variability was retained on all parameters (except inter-compartment clearance), and inter-occasion variability was significantly associated with relative bioavailability (*p <* 0.001) and mean transit time (*p <* 0.001).

Body weight as a fixed allometric function on all clearance and volume of distribution parameters improved the fit of the model significantly (ΔOFV = −419). Estimating the exponent on elimination clearance did not result in a significant drop in OFV (*p* > 0.001) compared to using a fixed exponent, and the estimated exponent was similar to the fixed value.

A categorical disease effect had a significant impact on elimination clearance, mean transit time, and central volume of distribution in the forward selection step (*p <* 0.05) and could be retained on mean transit time and elimination clearance in the backward elimination step (*p <* 0.001). However, during antimalarial treatment the patient usually recovers from the disease, so a disease effect on pharmacokinetics would be present only during the early assessments (day 1–3). Attempts to model a time-dependent disease effect failed, probably because of the low number of healthy volunteers (*n* = 50; 6.87%), of whom only 14 were dosed more than once. Thus, this covariate effect was not retained in the final model. Similarly, an exploratory analysis showed that disease could also have an effect on relative bioavailability, resulting in an increase in the exposure to piperaquine in healthy volunteers compared to patients. However, the disease effect was not retained in the final analysis, again because of the low number of healthy individuals. This needs to be addressed in future studies.

A 24% increase (*p <* 0.001) in relative bioavailability was observed between dose occasions, whereas the total daily milligram/kilogram dosage did not influence absorption. This finding is likely to be related to the recovery from malaria illness (and also increasing food intake) during the 3 d of treatment [25,27], as the systematic dose-occasion effect was estimated as close to zero in healthy volunteers. However, this covariate effect was not separated for patients and healthy volunteers in the final model due to the small number of healthy volunteers who had been dosed more than once (*n* = 14). Gender was found to affect the mean transit time significantly among malaria-infected non-pregnant adults, but this covariate relationship could not be retained in the more stringent backward step. Inclusion of a maturation factor produced only a minor improvement in model fit (ΔOFV = −3.29) among children below 5 y of age. However, inclusion of this covariate resulted in an estimated enzymatic maturation that reflected the biological maturation of infants’ piperaquine biotransformation pathways; therefore, this factor was included in the final model.

**Fig 3:**
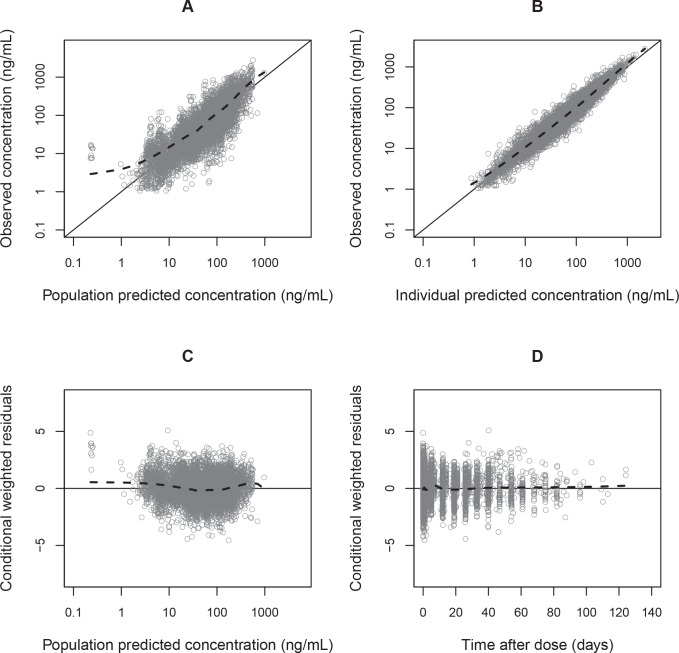
Basic goodness-of-fit plots for the final piperaquine model. Observed plasma piperaquine concentrations (from 11 clinical studies) plotted against population predicted concentrations (A) and against individual predicted concentrations (B). Conditional weighted residuals plotted against population prediction (C) and time (D). The solid line is the identity line, and the dashed line is the locally weighted least squares regression line.

**Fig 4:**
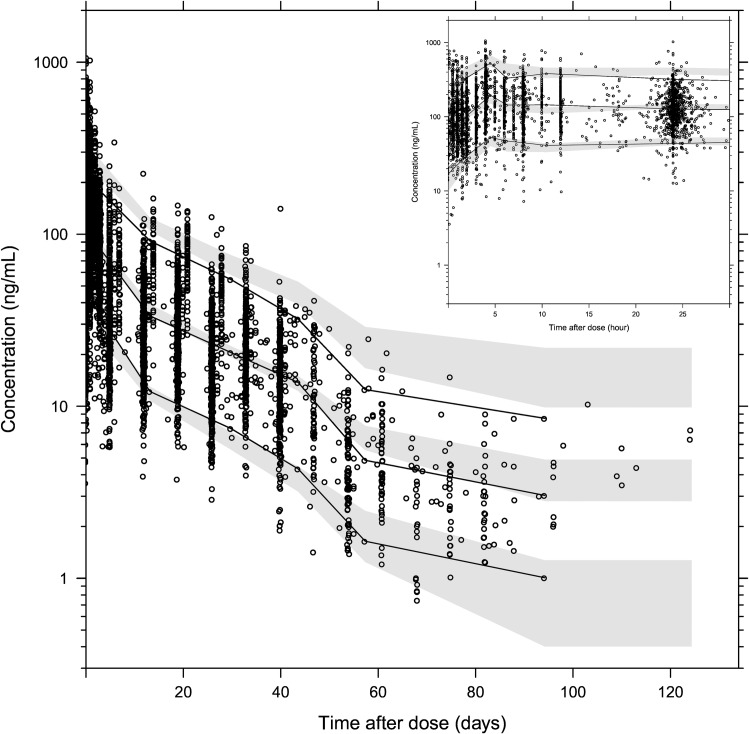
Prediction-corrected visual predictive check of the final population pharmacokinetic model of piperaquine. Based on 2,000 stochastic simulations. The insert shows the first 25 h after dosing. Open circles represent the observations, and solid lines represent the 5th, 50th, and 95th percentiles of the observed data. The shaded areas represent the 95% confidence intervals around the simulated 5th, 50th, and 95th percentiles.

Parameter estimates were reliable, with small relative standard errors (Table 3). Predicted secondary pharmacokinetic parameters (i.e., elimination half-life, maximum concentration, time to maximum concentration, total exposure, and day 7 plasma concentration) obtained from the final model are presented in Table 3. Calculated η shrinkages were high due to the sparseness of data in some of the individual studies, but the calculated ε shrinkage was low (14.6%). Goodness-of-fit diagnostics and the prediction-corrected visual predictive check (*n* = 2,000) demonstrated that the model described the observed data well (Figs 3 and 4). The visual predicted check showed a small model misspecification for capillary versus venous plasma concentrations, but overall the diagnostics supported using the developed model for stochastic simulations and dose optimisations.

**Table 3:** Final parameter estimates describing piperaquine population pharmacokinetics.

Parameter	Population Estimate^a^ [%RSE^b^] or Median (Minimum–Maximum)	95% CI^b^	Coefficient of Variation for IIV/IOV^a^ [%RSE^b^]	95% CI^b^	Shrinkage (Percent)^a^
CL/*F* (l/h)	55.4 [4.22]	51.2–60.6	27.9 [7.43]	23.7–31.7	38.1
*V*_C_/*F* (l)	2,910 [6.98]	2,540–3,340	67.0 [15.5]	49.7–89.4	46.9
*Q*_1_/*F* (l/h)	310 [8.03]	266–366	—	—	—
*V*_P1_/*F* (l)	4,910 [5.85]	4,390–5,510	24.0 [44.2]	1.35–40.4	71.4
*Q*_2_/*F* (l/h)	105 [4.98]	95.1–115	23.6 [15.6]	16.2–30.2	55.7
*V*_P2_/*F* (l)	30,900 [4.79]	28,300–34,200	34.7 [7.21]	29.6–39.4	46.2
MTT (h)	2.11 [4.54]	1.94–2.30	38.0 [15.8]/46.4 [9.36]	26.7–50.3/38.6–55.1	59.1/71.3
Number of transit compartments	2 *fix*	—	—	—	—
*F* (percent)	100 *fix*	—	41.4 [8.65]/53.5 [6.69]	34.0–48.2/46.1–60.2	31.1/59.7
RUV	0.115 [3.43]	0.108–0.123	—	—	14.6
Covariate relationships					
Scale (percent)	106 [7.24]	91.7–122	—	—	—
MF_50_ (years)	0.575 [13.6]	0.413–0.711	—	—	—
Hill_MF_	5.51 [29.6]	3.22–9.95	—	—	—
Dose_*F*_ (percent)	23.7 [17.8]	15.8–32.5	—	—	—
**Secondary parameters**					
*C*_MAX_ (ng/ml)	248 (24.3–1,070)				
*T*_MAX_ (h)	3.49 (1.13–10.0)				
*T*_1/2_ (d)	22.5 (9.15–52.3)				
AUC_∞_ (h × ng/ml)	28,800 (2,650–116,000)				
Day 7 concentration (ng/ml)	28.1 (2.35–115)				

Population estimates are given for a “typical” adult patient weighting 54 kg with acute falciparum malaria. CL/*F* is the apparent elimination clearance. *V*_c_/F is the apparent volume of distribution of the central compartment. *Q*_1_/*F* and *Q*_2_/*F* are the inter-compartment clearances between the central and the peripheral compartments. *V*_P1_/*F* and *V*_P2_/*F* are the apparent volumes of distribution of the peripheral compartments. MTT is the mean transit time of the absorption. Number of transit compartments is the number of transit compartments used in the absorption model. *F* is the relative bioavailability. RUV is the variance of the unexplained residual variability. Scale is the difference between venous and capillary predictions. MF_50_ is the maturation age (in years) to reach 50% of the full elimination clearance. Hill_MF_ is the Hill function in the maturation equation, with an upper limit of 10. Dose_*F*_ represents the increase in relative bioavailability between each dosing occasion. *C*_MAX_ is the maximum concentration. *T*_MAX_ is the time after dose to reach the maximum concentration. *T*_1/2_ is the terminal elimination half-life. AUC_∞_ is the area under the concentration–time curve from time 0 to infinity. Day 7 concentration is the venous plasma concentration at day 7 after dosing. Coefficients of variation for inter-individual variability (IIV) and inter-occasion variability (IOV) were calculated as 100 × (e^variance^ − 1)^1/2^. Relative standard errors (%RSE) were calculated as 100 × (standard deviation/mean). The 95% confidence intervals are given as the 2.5 to 97.5 percentiles of bootstrap estimates. Secondary parameters were calculated after the last dose.

^a^Based on population mean values from NONMEM.

^b^Based on 658 successful stratified bootstrap runs (out of 1,000).

### Dose Optimisation

**Fig 5:**
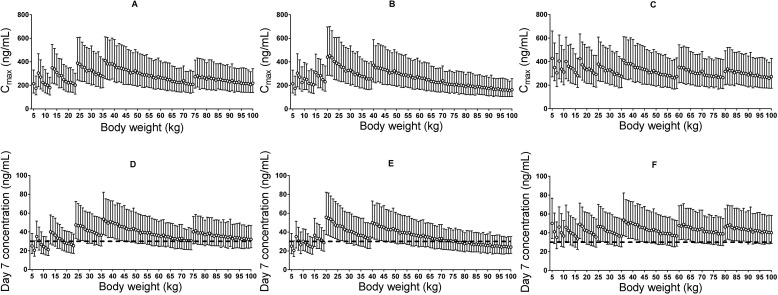
Stochastic simulations of dose regimens. Maximum plasma piperaquine concentration (A–C) and day 7 plasma piperaquine concentration (D–F) after Sigma-Tau’s recommended dosing (left panels), Beijing Holley-Cotec’s recommended dosing (middle panels), and the revised dose regimen (right panels). Circles represent the median values, and vertical lines represent the 25th to 75th percentiles of simulated concentrations. The dashed line indicates the previously defined venous plasma piperaquine day 7 concentration threshold value for therapeutic success of 30 ng/ml [35].

The derived pharmacokinetic model predicted lower plasma piperaquine exposures in small children (5–24 kg) and adults with body weight between 60 and 75 kg (or between 60 and 100 kg if following the dose recommendation from Beijing Holley-Cotec) compared to other adult patients after dihydroartemisinin-piperaquine administration following the manufacturers’ recommended dose regimens. Simulated median (interquartile range) day 7 plasma concentration was 29.4 (19.3–44.3) ng/ml in small children (<25 kg) compared to 38.1 (25.8–56.3) ng/ml in larger children and adults (≥25 kg), with Sigma-Tau’s recommended dose regimen. The revised dose regimen proposed here is expected to achieve predicted exposures at all body weights comparable to that currently seen in a typical adult after appropriate standard treatment (Table 2; Fig 5). Simulated maximum plasma piperaquine concentrations after manufacturers’ dosing and the new optimised dose regimen are presented in Fig 5. The minimum and maximum piperaquine dosages of the three evaluated dose regimens are summarised in Table 2. It is important to note that the predicted maximum plasma piperaquine concentrations with the optimised regimen (upper 75th percentile of approximately 600 ng/ml) are not higher than those observed with the manufacturers’ regimens.

## Discussion

This study developed and validated a population pharmacokinetic meta-model to describe the pharmacological properties of piperaquine and the influence of demographic and clinical covariates. Body weight influenced clearance and volume parameters significantly, resulting in lower piperaquine exposures in small children compared to larger children and adults after administration of the manufacturers’ currently recommended dose regimens. The final model was used to develop a revised dose regimen of dihydroartemisinin-piperaquine that is expected to provide therapeutic piperaquine exposures safely in all patients, including in small children with malaria. This could improve the treatment of malaria in small children.

Dihydroartemisinin-piperaquine is an excellent fixed-dose ACT that has shown consistently good efficacy in patients with uncomplicated *P*. *falciparum* malaria infection [4–8], but cumulative evidence shows that dosing could be improved [19]. Under-dosing increases the risk of treatment failure and therefore drives the development of resistance. Piperaquine resistance developed in China, where piperaquine monotherapy was used for prevention and treatment between 1978 and 1994, and has emerged again recently in Cambodia on a background of artemisinin resistance [53–55]. To maximise its therapeutic lifespan, it is essential to optimise dihydroartemisinin-piperaquine recommended dose regimens, which would lower the risk of treatment failure and reduce the selective pressure for the development of resistance. The present study used a pooled pharmacokinetic modelling approach to evaluate dihydroartemisinin-piperaquine treatment and presents an optimised dose regimen that should improve the treatment of uncomplicated *P*. *falciparum* malaria.

A large WWARN pooled analysis of the clinical efficacy of dihydroartemisinin-piperaquine in 7,072 patients enrolled in 26 studies highlighted a significant risk of recrudescent malaria in young children (1–5 y of age) [19]. Younger children typically have the highest treatment failure rates in meta-analyses of antimalarial treatments [19,21,56,57], usually attributed to relative lack of immunity [20]. But lower drug exposure is another possible contributor. The previously published pooled analysis demonstrated that the milligram/kilogram piperaquine dosage administered was a significant predictor of treatment failure, with the risk of recrudescence increased by 13% (95% CI 5.0%–21%) for every 5-mg/kg decrease in dosage on day 42 [19]. Previous clinical studies as well as pharmacokinetic-pharmacodynamic modelling show that children achieved a lower plasma exposure to piperaquine, compared to adults, after equivalent weight-based (mg/kg) piperaquine dosage [24,30,35]. Taken together, these findings suggest the need to increase dose regimens of dihydroartemisinin-piperaquine for small children.

Several studies have investigated the pharmacokinetic properties of piperaquine [6,10,23–25,27,29,30,32,35,40,58–60]. However, the present study is, to our knowledge, the largest analysis to date, incorporating data from 8,776 pharmacokinetic samples from 728 individuals from different target populations and continents. Our results can be used to inform evidence-based optimised treatment in vulnerable young children with malaria, who account for an estimated 78% of malaria-related deaths [1].

### Pharmacokinetics of Piperaquine

A three-compartment disposition model with a transit compartment absorption model was found to describe the pooled data adequately, which is in accordance with other recent studies [24,25,27,29,30]. Inter-occasion (within-individual) variability had a significant impact on both relative bioavailability and the mean transit time of the absorption, indicating a large degree of variability not just among patients but also between dose occasions for a specific patient. The final model showed satisfactory goodness-of-fit diagnostics and high precision in parameter estimates. Estimated parameters were also comparable to those found in previous studies [22,24,25,27,29]. The visual predictive check showed a small model misspecification for the capillary versus venous plasma data, probably because capillary measurements were performed only in studies of children with malaria, whereas the overall model is based on a more diverse dataset. The model simulation therefore included more variability compared to the observed data.

In agreement with previous studies [25,27], piperaquine absorption in patients with malaria could be characterised further with a categorical covariate that increased the relative bioavailability by 24% with each subsequent dose within a single course of treatment. This increase in relative bioavailability may result from the improved gastrointestinal function and food intake that accompanies clinical recovery. However, a disease effect on bioavailability could not be evaluated given the few healthy volunteers (*n* = 14) who received more than one dose.

Most drug measurement data in children were based on capillary samples, compared to venous plasma sampling in adult patients. A constant scale factor to convert between capillary and venous plasma piperaquine measurements was therefore required to allow simultaneous modelling of all individual patient data. The estimated capillary piperaquine concentrations were 106% higher than venous piperaquine concentrations, which is a greater difference than that reported previously [58]. However, this conversion factor resulted in a good description of both venous and capillary observations. An additional advantage of a simultaneous approach is that the model can be used to predict drug exposures from any sampling technique and therefore enables literature comparisons. The exact mechanism underlying this matrix-dependent difference cannot be elucidated from the data pooled in this analysis and needs further evaluation.

Metabolic enzymes mature during the first years of life in a way that cannot be explained by an allometric function of body weight [61]. Most hepatic enzymes reach 70% to 100% maturation during the first 12 mo of life [62]. Sambol et al. investigated age as a nonlinear covariate (in addition to weight) and identified that, both covariates taken together, 6-mo-old infants have approximately half the clearance of 2-y-old children [30]. To investigate this further, the present study assessed age as a maturation function on piperaquine elimination clearance. The inclusion of a maturation function improved the model fit. Although the improvement was not statistically significant, the factor was kept in the final model to reflect the known changes in biotransformation pathways that occur as the infant grows. More information is needed on piperaquine disposition in the first 2 y of life.

A malaria disease effect was investigated but not retained in the final model given the few healthy volunteers who received more than one dose (*n* = 14). However, the preliminary results suggest that healthy patients have higher maximum concentrations and higher exposures to piperaquine. This is an important caveat to the revised dosing recommendations—they apply to the treatment of malaria. More information is needed on the pharmacokinetic properties of 3-d dihydroartemisinin-piperaquine regimens in healthy individuals. This is particularly important as dihydroartemisinin-piperaquine is also being used in mass treatment campaigns where most of the recipients are healthy.

Pregnancy was not evaluated in this analysis, since only 4.9% (*n* = 36) of the patients in the study population were pregnant. However, pregnancy is expected to have a limited influence on the pharmacokinetic model presented here. Separate pharmacokinetic evaluations of the two studies that included pregnant women concluded that there were no differences in total piperaquine exposures between pregnant and non-pregnant patients [27,29].

Young children in areas of high malaria transmission are at increased risk of developing life-threatening severe malaria. Indeed, most of the deaths from malaria occur in African children. Partially protective immunity that also boosts parasite elimination after treatment only develops after repeated infections. It is important that children receive effective treatments at the doses needed to provide adequate exposure to all antimalarial drug components, but particularly the artemisinin partner in the case of an ACT. Sub-therapeutic exposure also increases the risk of drug resistance. The Sigma-Tau-recommended piperaquine dosage targets a daily dosage of 18 mg/kg, with a range between 13 and 27 mg/kg at different body weights (Table 2), while the dosage recommended by Beijing Holley-Cotec ranges from 9.6 to 32 mg/kg. A constant weight-based (mg/kg) dosage target would be appropriate only if there were a linear relationship between drug exposure and body weight, but physiological processes do not scale linearly with body weight [30,61,63]. Body weight has been identified as an important predictor of piperaquine exposure in previous studies [22,24,25,29], as confirmed in this study. Consequently, children achieve a lower drug exposure compared to adults after a standard target daily dosage of 18 mg/kg, which increases their risk of treatment failure and could shorten the useful therapeutic life of dihydroartemisinin-piperaquine [19]. This has potentially important consequences for therapeutic outcome and the development of drug resistance. It likely contributes to the >3-fold higher risk of recrudescence observed in children aged 1–5 y (hazard ratio 3.71; 95% CI 1.66–8.26; *p* = 0.002) compared to patients >12 y of age [19]. Consequently, in order to achieve exposure similar to that of adults, small children need higher doses of piperaquine than currently recommended by the manufacturers.

### Dose Optimisation

The pooled analysis presented here is, to our knowledge, the largest pharmacokinetic analysis of piperaquine to date and incorporates data collected in phase III clinical trials and post-marketing studies in a large variety of populations, resulting in a greater power to identify important pharmacokinetic differences between key target populations. The final model showed adequate predictive performance, demonstrating its suitability for dose optimisation simulations. The final pharmacokinetic model was therefore used to simulate piperaquine exposures and maximum piperaquine concentrations at different body weights using the dose regimens recommended by the manufacturers (Sigma-Tau and Beijing Holley-Cotec) and was used to develop an evidence-based improved dose regimen for small children.

Our simulations (Fig 5) show that both small children and adults with body weights between 60 and 75 kg for the Sigma-Tau–recommended dose regimen (or between 60 and 100 kg for the Beijing Holley-Cotec–recommended dose regimen) achieve lower plasma piperaquine exposures than typical adult patients (35–65 kg) with acute falciparum malaria after standard dosing (Table 2). The revised dosing scheme (Table 2) is predicted to achieve equivalent plasma piperaquine exposures in all patient groups, including small children and larger adults, without risking higher maximum piperaquine concentrations (Fig 5). A previously pooled efficacy analysis of dihydroartemisinin-piperaquine treatment [19] showed that a total minimum piperaquine dosage of 59 mg/kg would result in successful treatment in 95% of small children. Our revised dose scheme proposes a total minimum dosage of 64 mg/kg for children weighing 5–15 kg (Table 2), compared to the minimum dosage of 40 mg/kg recommended by the manufacturers. This adjustment would ensure similar plasma piperaquine exposure across all weight groups and, most importantly, would improve the treatment of small children.

Most ACTs have a target artesunate dosage of 4 mg/kg/d according to WHO guidelines for the treatment of malaria. This corresponds to a dihydroartemisinin dosage of 2.96 mg/kg/d (based on molar equivalents). However, the current manufacturer-recommended dihydroartemisinin dosage ranges between 1.62 and 3.33 mg/kg/d for Sigma-Tau, and between 1.20 and 4.00 mg/kg/d for Beijing Holley-Cotec, giving the lowest dosage of the artemisinin component of all WHO-recommended ACTs. As dihydroartemisinin-piperaquine is a fixed-dose combination, the increased piperaquine doses recommended by this analysis will also increase the dihydroartemisinin dosage, while remaining within the 2–10 mg/kg target range recommended by WHO. Increasing both dihydroartemisinin and piperaquine concentrations should contribute to a more effective therapy by reducing the residual parasite biomass remaining on day 3, and reduce the risk of recrudescence and reinfection, thereby potentially prolonging the useful therapeutic life of dihydroartemisinin-piperaquine.

Piperaquine prolongs ventricular repolarisation, and this is reflected in electrocardiographic QT prolongation. Manning et al. [64] stopped a recent study in healthy volunteers given a 50% higher than recommended daily dose of piperaquine for 2 d because four volunteers had potentially unsafe QT prolongation (>500 ms). However, that study used the automated electrocardiograph reading, mostly likely resulting in a reported QU interval instead of the correct manual QT reading, which would be substantially shorter. Furthermore, increasing the piperaquine dose by 50% in these healthy volunteers resulted in very high maximum plasma piperaquine concentrations, with a mean value of 1,750 ng/ml [64], compared to a predicted median maximum concentration of 310 ng/ml following the suggested optimised dose regimen (Fig 5). Small children receiving higher body-weight-based daily doses of piperaquine, after dose adjustment, are not expected to achieve higher maximum piperaquine concentrations than a typical non-pregnant adult patient given the manufacturer’s recommended dose regimen (Fig 5). Thus, maximum plasma piperaquine concentrations after optimised dosing are not expected to increase the risk of cardiac adverse events. However, the safety and efficacy of this suggested revised dosing will need to be evaluated prospectively. Such prospective studies could also assess whether more pragmatic dose regimens with fewer body weight bands could be achieved safely.

In the context of antimalarial drug registration for uncomplicated *P*. *falciparum* malaria, patients recruited for phase II or phase III trials usually exclude important sub-populations such as infants, pregnant women, and patients with co-morbidities (e.g., malnutrition, co-infections). Thus, these sub-populations are unlikely to be represented in sufficient numbers to draw a conclusion on their optimal dosing at the time of the initial registration of the drug with a medicines regulatory authority. Pooled analyses of individual patient data accumulated in the post-marketing phase are needed to allow dose optimisation for such vulnerable target population groups, as these are the populations that carry the highest malaria morbidity and mortality rates.

However, limited data were available in infants (≤1 y of age), pregnant women with malaria, patients with co-morbidities, and healthy individuals for this pooled meta-analysis. Thus, the pharmacological properties of piperaquine could not be assessed reliably in these groups within the present analysis. Prospective pharmacological studies are urgently needed to address potential differences in these sub-groups. Other study limitations are the lack of general safety data, and the lack of data from large monotherapy piperaquine trials performed in China between 1978 and 1994.

In conclusion, suboptimal plasma piperaquine exposures in small children given the current manufacturers’ recommended dose regimens were confirmed in this pooled pharmacokinetic analysis. In addition, low exposure in adults with body weights of 60–100 kg (depending on dose regimen) was also detected. Pharmacokinetic analysis was used to derive an optimised antimalarial dose regimen. It is essential that currently used antimalarial treatments are optimised in the post-registration phase so that all patient groups achieve similar drug exposures, and thus an equal chance of being cured. This optimisation would also reduce the selective pressure for the development of resistance, thus slowing the development of drug resistance and prolonging the useful therapeutic life of dihydroartemisinin-piperaquine. It is essential that currently available antimalarials remain effective until novel treatments can be produced to overcome artemisinin resistance. This evidence-based improved dose regimen has been adopted by WHO in their recently published guidelines for the treatment of malaria [2].

## Supporting Information

S1 PRISMA IPD Checklist PRISMA checklist. (DOCX)

## Abbreviations

ACT: artemisinin-based combination therapy
OFV: objective function value
WHO: World Health Organization
WWARN: WorldWide Antimalarial Resistance Network
